# Disruptions of cell signaling pathways in myotonic dystrophy type 1 skeletal muscle, their pathogenic impact, and potential for combinatorial therapeutics

**DOI:** 10.1016/j.jbc.2026.111219

**Published:** 2026-01-30

**Authors:** Aymeric Ravel-Chapuis, Shatha A. Atieh, Chimène Fahmi, Bernard J. Jasmin

**Affiliations:** 1 Faculty of Medicine, School of Pharmaceutical Sciences, University of Ottawa, Ottawa, Ontario, Canada; 2 Faculty of Medicine, Department of Cellular and Molecular Medicine, University of Ottawa, Ottawa, Ontario, Canada; 3 Faculty of Medicine, Eric Poulin Centre for Neuromuscular Disease, University of Ottawa, Ottawa, Ontario, Canada

**Keywords:** myotonic dystrophy, cell signaling, skeletal muscle, therapeutics

## Abstract

Myotonic dystrophy type 1 (DM1) is caused by a CUG expansion located in the 3′ UTR of dystrophia myotonica protein kinase mRNAs. The pathogenic model underlying DM1 implicates the accumulation of mutant dystrophia myotonica protein kinase transcripts in nuclei where they form toxic RNA foci. This, in turn, disrupts the availability of RNA-binding proteins causing widespread missplicing of mRNAs. Over the years, multiple signaling pathways have also been reported to be disrupted in DM1, especially in skeletal muscle. Here, we focus on several pathways including protein kinase R, PKC, glycogen synthase kinase 3β, Akt-mTOR, AMP-activated protein kinase, TWEAK-Fn14 and NF-κkB, and calcineurin-NFAT. We describe the individual effects of these signaling disruptions on multiple muscle functions and characteristics, and we also present an overview of their cumulative impact. Based on the available literature, dysregulation of signaling in muscle jointly results in global perturbations in protein synthesis and degradation, muscle repair, mitochondrial biogenesis, energy metabolism, and inflammation. The fact that pharmacological, physiological, and transgenic approaches targeting these pathways corrected defects observed in DM1 muscle provides a strong rationale for therapeutic intervention. These pathways can be targeted individually or through combinatorial treatments involving two or more agents. Based on the impact of these signaling pathways on multiple aspects of the DM1 muscle phenotype, therapeutically targeting these disruptions is becoming increasingly attractive and represents a critical area for additional research in the quest to slow or reverse muscle dysfunction in DM1.

Myotonic dystrophy type 1 (DM1) is a multisystemic disorder that primarily affects skeletal muscle but also involves multiple other organs, including the heart, gastrointestinal tract, central nervous system (CNS), eyes, and endocrine system (1). The global prevalence of DM1 is approximately 0.5 to 18.1 per 100,000 individuals, although it can be significantly higher, reaching up to 158 per 100,000 in specific regions, as in the Saguenay-Lac-St-Jean area in Quebec, Canada, due to a founder effect (2, 3). DM1 is caused by an unstable expansion of CUG trinucleotide repeats (CUG^exp^) in the 3′ untranslated region (UTR) of the dystrophia myotonica protein kinase (*DMPK*) mRNA (4, 5). The length of this repeat expansion correlates with disease severity (6). While asymptomatic individuals typically carry between 5 to 37 repeats, mild late-onset, classic adult-onset, and congenital forms of the disease are associated with the expansions of 50 to 150, 100 to 1000, and exceeding 1000 repeats, respectively (7).

Due to the nature of the underlying mutation, DM1 is seen as an RNA-mediated disorder. Indeed, the prevailing pathogenic model stipulates that mutant *DMPK* mRNAs carrying expanded CUG repeats accumulate in the nucleus as RNA foci which sequester and disrupt the function of multiple RNA-binding proteins (RBPs) involved in RNA splicing. This leads to widespread misregulation of mRNA alternative splicing. Among the RBPs affected, Muscleblind-like protein 1 (MBNL1) has been most extensively studied (8). However, several other RBPs are also implicated in the disease mechanism, including CUGBP Elav-like family member 1 (CELF1 or CUGBP1) (9, 10), Staufen1 (11, 12), heterogeneous nuclear ribonucleoproteins, and DEAD-Box Helicases (13, 14, 15, 16, 17, 18, 19). Collectively, these disruptions form the basis of the DM1-associated spliceopathy, a molecular hallmark of the disease with numerous aberrant splicing events linked to specific clinical manifestations. For example, misregulation of *INSR*, *CLCN1*, and *SCN5A* mRNA splicing has been associated with insulin resistance, myotonia, and cardiac conduction defects, respectively (9, 20, 21, 22, 23, 24).

In line with this RNA toxicity model, early therapeutic strategies have mostly focused on the following: (i) reducing *DMPK* mRNA levels or preventing its aggregation using antisense oligonucleotides, siRNAs, or CRISPR/Cas-based approaches; (ii) blocking the sequestration of MBNL1 by CUG^exp^ *via* antisense oligonucleotides or small molecules; (iii) restoring the expression or function of dysregulated RBPs; and (iv) correcting aberrant splicing using exon-skipping or splicing-modulation strategies. Despite encouraging results in preclinical models, none of these RNA toxicity-targeted approaches have yet translated into an effective or clinically approved treatment for DM1 patients (25, 26, 27, 28, 29, 30).

In recent years, multiple signaling pathways have been found to be disrupted in DM1, particularly in skeletal muscle. These alterations contribute not only to the symptoms of the disease but also to the core pathophysiology. In this review, we summarize current insights into the signaling pathways altered in DM1 skeletal muscle and examine their individual contribution to the disease phenotype while also providing an overview of their cumulative and interconnected impact. Collectively, these signaling pathways offer new avenues for therapeutic interventions including combinatorial approaches targeting simultaneously more than one pathway.

## Disrupted cellular processes and altered signaling pathways in DM1

### Cellular stress and RNA toxicity in DM1

#### PKR signaling

**Figure 1:**
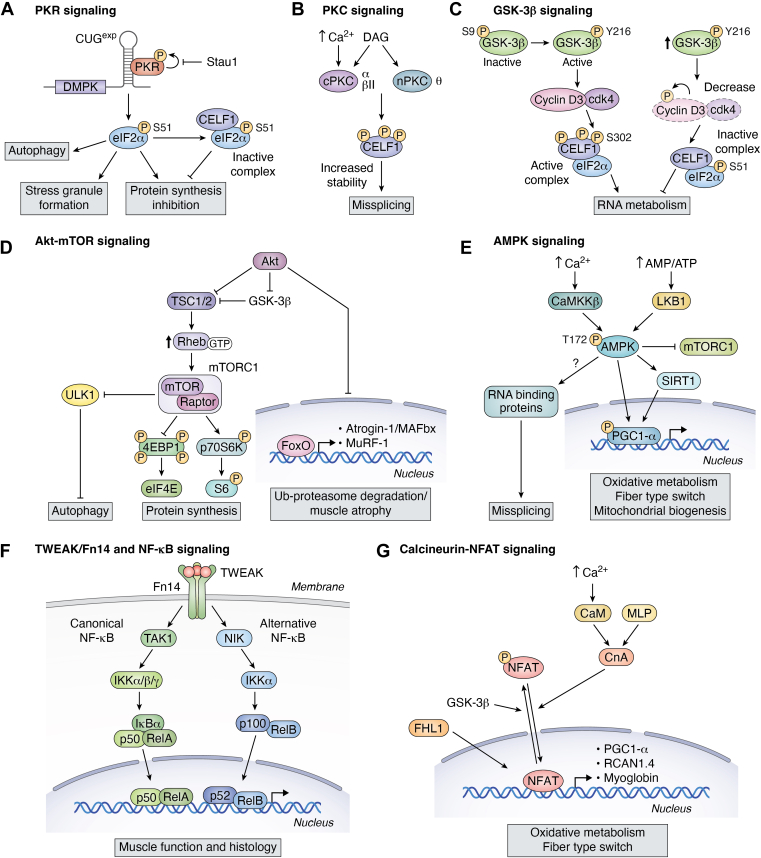
**Schematic representation of the altered signaling pathways in DM1.** Panels *A to G* illustrate the major signaling pathways disrupted in DM1 muscle. Question mark (?) in the figure represents potential interactions that have not yet been elucidated.

Protein kinase R (PKR) is a serine/threonine kinase activated by dsRNA and is best known for its role in antiviral immunity. Upon binding to dsRNA, PKR undergoes autophosphorylation, resulting in its activation. Activated PKR subsequently phosphorylates eukaryotic initiation factor 2α (eIF2α) on serine 51, thereby inhibiting translation initiation and suppressing global protein synthesis (Fig. 1*A*) (for review see (31)). This translational inhibition promotes the formation of stress granules (SGs), which serve as transient storage sites for stalled preinitiation complexes during cellular stress. In addition to its role in translational control, PKR also contributes to autophagy, a critical cellular process involved in the degradation and recycling of damaged organelles, misfolded proteins, and intracellular pathogens through autophagosome-lysosome fusion.

The CUG^exp^ present in the 3′UTR of *DMPK* mRNA folds into a dsRNA hairpin structure, forming a large platform for protein interactions within the nucleus (32). These CUG^exp^ structures are known to bind and sequester MBNL1 (see above), thereby reducing the availability of functional MBNL1 and altering MBNL1-dependent alternative splicing events (8). In search of additional proteins capable of binding CUG^exp^, PKR was found to directly interact with and become activated by CUG^exp^ *in vitro*, suggesting that expression of these toxic hairpin-forming RNAs in DM1 cells could potentially activate PKR and initiate downstream signaling (33). However, subsequent studies demonstrated that PKR does not colocalize with CUG^exp^ RNA foci in DM1 neuronal cells or in skeletal muscle from HSA^LR^ mice (a muscle-specific DM1 mouse model expressing approximately 250 CTG repeats in the 3′UTR of the human skeletal actin (HSA) transgene (34)), suggesting that PKR is not stably sequestered by CUG^exp^ mRNAs (35, 36). Although there are no recent studies that have further clarified the role of PKR in the DM1 context, these earlier works nonetheless, established CUG^exp^ RNA foci as dynamic structures. Notably, expanded transcripts can also exist outside nuclear foci (37), underscoring the transient interactions between PKR and both free and aggregated CUG^exp^ RNA. Such interactions cannot be excluded in the DM1 pathogenesis and may therefore account for PKR activation observed in DM1 cells.

Although PKR does not appear to be sequestered by CUG^exp^ transcripts, elevated levels of both PKR and phospho-eIF2α have been observed in DM1 patient-derived myoblasts (Table 1), supporting a model in which PKR activation contributes to SG dynamics in DM1 (38). Specifically, increased PKR activity in DM1 cells leads to enhanced phosphorylation of eIF2α, which is subsequently recruited into SGs. Within these granules, phospho-eIF2α binds unphosphorylated CELF1 to form an inactive CELF1–eIF2α complex, thereby repressing the translation of specific mRNA targets (38).

**Table 1:** Signaling pathways altered in DM1

Signaling pathway	Status in DM1	Model	Consequences for DM1	References
PKR	- Increased protein and activity levels - Increased phospho-eIF2α - Not stably sequestered by CUG^exp^ foci	- Patient-derived DM1 myoblasts - HSA^LR^ mice - DM1 neuronal cells	- Stress response through eIF2α - Translational repression - SG formation - Altered CELF1 translation	(35, 36, 38, 39)
PKC	- PKC-α and PKC-βII activated in EpA960/MCM and in patient-derived skin fibroblasts - PKC-θ increased in DM5 and DM200 mice	- Patient-derived skin fibroblasts - Human DM1 heart tissues - EpA960/MCM - DM5 mice - DM200 mice	- Phosphorylates and stabilizes CELF1 - Induces splicing misregulation	(52, 62, 64)
GSK-3β	- Increased GSK-3β - Increased phospho-GSK-3β (Tyr-216) - Decreased phospho-GSK-3β (Ser-9)	- HSA^LR^ mice - DMSXL mice - DM1 patient muscles and neural stem cells	- Promotes CELF1 dysregulation *via* cyclin D3 degradation - Disruption in splicing, RNA metabolism and translation	(38, 47, 70, 71, 72)
Akt-mTOR	- Decreased mTOR activity (p-Ser-2448) - Variable Akt and p70S6K/S6 phosphorylation	- Patient-derived DM1 myotubes - Human and iPSC-derived satellite muscle cells - HSA^LR^ mice	- Increased autophagic activity	(71, 83, 84)
AMPK	- Decreased phospho-AMPK/AMPK in HSA^LR^ - Increased AMPK activity in CUG960	- HSA^LR^ mice - CUG960 mice - DM1 fibroblasts/myotubes	- Decrease in mitochondrial biogenesis - Energy imbalance - Compromised metabolic processes	(84, 94, 100)
TWEAK-Fn14- NF-κB	- Increased Fn14 expression - Increased p-p65 and p52 levels	- DM5 mice - DM200 mice - DM1 patient muscle biopsies	- Activates NF-κB; drives inflammation, atrophy, fibrosis - Exacerbates muscle degeneration	(121)
Cn-NFAT	- Upregulated	- HSA^LR^ mice	- Upregulates oxidative genes - Increases in muscle fiber size - Protective role in skeletal muscle	(139)

Supporting a role of PKR in RNA toxicity, pharmacological inhibition of PKR using the imidazole oxindole compound C16, as well as the knockdown of its upstream activator PACT, significantly reduced the number of RNA foci in DM1 patient-derived fibroblasts. This indicates that PKR modulates RNA toxicity at least in culture (39). Further studies using selective PKR inhibitors (40, 41, 42, 43) in DM1 mouse models could help clarify the role of PKR in DM1 pathogenesis. Genetic deletion of PKR in HSA^LR^ mice (HSA^LR^/PKR^−/−^ mice) revealed that PKR is not essential *per se* for the DM1 pathogenesis (36) (Table 2). However, this finding does not completely rule out the participation of PKR in DM1 pathogenesis, as compensatory pathways may mask its effect in knockout models.

**Table 2:** Genetic manipulations in DM1 mouse models

Signaling pathway	Transgenic mouse models	Functional effects on DM1 phenotype	References
PKR	HSA^LR^/PKR^−/−^ mice	No improvements in DM1 muscle histology	(36)
PKC	DM5/*Prkca*^*−/−*^*/Prkcb*^*−/−*^ mice	No improvements in histology or splicing as CELF1 upregulation resists	(62)
TWEAK-Fn14- NF-κB	DM5/Fn14^−/−^ mice	Reduction in muscle damage; improved muscle function; no effect on myotonia, cardiac conduction defects, and alternative splicing	(121)
	DM5/Tweak^−/−^ mice	Improved muscle function and histology	(122)

Our laboratory previously demonstrated that the RBP Staufen1 is upregulated in skeletal muscle from both HSA^LR^ mice and DM1 patients (11), where it acts as a disease modifier (44). Elevated levels of Staufen1 contributes to aberrant alternative splicing (11, 12), impairs SG formation (45), and promotes muscle atrophy (46, 47) in the DM1 context. Given that Staufen1 binds to PKR and inhibits its autophosphorylation (48, 49), it appears plausible that Staufen1 upregulation may thus have dual roles: in one context, it may elicit muscle atrophy through its effects on splicing and SG dynamics, whereas in another, it may exert a compensatory effect by attenuating PKR activation thereby mitigating the impact of cellular stress associated with DM1. While there is little direct evidence to date for the role of PKR in DM1 beyond *in vitro* studies, this potential interplay suggests that Staufen1 exerts context-dependent effects in DM1, with both detrimental and protective effects. As a result, further investigation of the Staufen1–PKR axis *in vivo* appears warranted to clarify whether this potential inhibitory effect on PKR is valid and pathologically significant in the DM1 context.

### CELF1-driven cellular dysfunction in the DM1 context

#### PKC signaling

PKCs are a family of serine/threonine kinases divided into three groups based on their homology and mode of regulation (Fig. 1*B*). Conventional PKCs (PKC-α, PKC-βI, PKC-βII, and PKC-γ) are activated by both diacylglycerol (DAG) and calcium due to the presence of DAG- and calcium-binding domains. Novel PKCs (PKC-δ, PKC-ε, PKC-η, and PKC-θ) require DAG for activation but are calcium-independent. Atypical PKCs (PKC-ζ, PKC-ι, and PKC-μ) are insensitive to both DAG and calcium (for review, see (50, 51)).

Several PKC isoforms have been implicated in the upstream regulation of CELF1, highlighting their potential involvement in the molecular pathology of DM1 (52). CELF1 was initially identified for its ability to bind to CUG repeats (53, 54). Although CELF1 does not colocalize with nuclear CUG^exp^ RNA foci (8, 32), its steady-state levels are increased in DM1 myoblasts, skeletal muscle, and cardiac tissues (9, 10, 55), as well as in transgenic mice expressing CUG^exp^-containing transcripts (56, 57) (Table 1). Interestingly, CELF1 upregulation also occurs in the skeletal muscle of an inducible DM1 mouse model. This mouse model expresses only five CUG repeats in the 3′UTR of the *DMPK* mRNA downstream of a GFP transcript following induction with doxycycline (inducible DM5 model) (58). Elevated CELF1 levels contribute to splicing misregulation of key DM1-relevant transcripts including *Clcn1, Tnnt2* (59), and *Ca*_*V*_*1.1* (22). Importantly, the increase in the steady-state levels of CELF1 was shown to be dependent on its hyperphosphorylation (52).

Increased levels of both total and phosphorylated CELF1 have been reported in skeletal and cardiac muscle of DM1 mice, as well as in human DM1 heart tissue (52). In cardiac tissues, these changes are accompanied by elevated PKC expression. Genetic approaches further support the role of PKCs in CELF1 regulation. Depletion of PKC-α in H9c2 cardiac cells impairs CELF1-dependent splicing (60), while knockdown of PKC-βII and PKC-θ modulates MBNL1-dependent SERCA1 splicing in HEK293 cells (61).

To further assess the role of these PKC isoforms in DM1, total levels of PKC-α and PKC-θ, as well as phosphorylated PKC-α/βII were measured from skeletal muscles of a DM1 mouse model (inducible DM5 mice) (62). These levels were found to be similar between DM1 mice and control groups (uninduced DM5 mice). Thereafter, a DM5 mouse model lacking both PKC-α and PKC-β (DM5/*Prkca*^−/−^/*Prkcb*^−/−^ mice) was generated to better understand the functional contribution of PKC isoforms on RNA toxicity (Table 2). These mice showed no differences in muscle histology (central nucleation) or splicing, indicating that the DM1 phenotype is not reliant on these isoforms (62). Moreover, the combined genetic ablation of PKC-α and PKC-βII in DM5 mice did not eliminate CELF1 upregulation, suggesting functional redundancy or compensation by other kinases, such as PKC-θ (62) (Table 2). Interestingly, phosphorylated PKC-θ was significantly increased in induced DM5 and DM200 mice, expressing 200 CTG repeats, compared to their respective controls (62). This suggests that increased PKC-θ activity drives CELF1 hyperphosphorylation, contributing to the DM1 phenotype. These findings indicate that PKC-θ plays a crucial role in regulating key biological processes related to the DM1 pathology and may thus be an important therapeutic target to explore in DM1.

Conventional PKC-α and PKC-βII have also been shown to be activated in patient-derived skin fibroblasts, in human DM1 heart tissues, and in the EpA960/MCM inducible mouse model expressing a *Dmpk*-CUG960 transcript (52) (Table 1). These isoforms can directly phosphorylate CELF1 *in vitro*, leading to its stabilization (52). However, the precise phosphorylation sites on CELF1 remains to be identified, and variability in CELF1 phosphorylation is observed in *biceps brachii* biopsies from DM1 patients (63).

Treatment of EpA960/MCM mice with the PKC inhibitor Ro-31-8220 for 24 days prevents PKC activation and abolishes CELF1 hyperphosphorylation and upregulation (64) (Table 3). This treatment leads to improved lifespan and amelioration of cardiac conduction defects and contractile abnormalities (64). A second inhibitor, Ro-32-0432, yielded similar but milder effects, reinforcing the therapeutic potential of targeting PKC in DM1 (64). Accordingly, Ro-31-8220 treatment corrected CELF1-dependent splicing defects in the heart of EpA960/MCM mice (64). Together, these data demonstrate that PKC inhibition prevents CELF1-mediated aberrant splicing.

**Table 3:** Pharmacological and physiological modulation of signaling pathways in DM1 mouse models

Signaling pathway	Modulators	Mouse model	Molecular effects	Histological/Functional effects	References
PKC	Inhibitors: - Ro-31-8220 (i.p., 6 mg/kg/d, 24 days) - Ro-32-0432 (i.p., 6 mg/kg/d)	EpA960/MCM	- Downregulation of CELF1 hyperphosphorylation - Corrects CELF1-splicing defects	- Improvement in cardiac symptoms and contractile abnormalities	(64)
GSK-3β	Inhibitors: - Lithium (Chow, 0.24%, 2 weeks) - TDZD-8 (i.p., 10 mg/kg, 2–7 days) - BIO (i.p., 3.6 μg/g/2d, 6 weeks) - Indirubin (i.p., 2.0 mg/kg/2d, 6 weeks) - TG (oral gavage, 0.025, 0.05, and 0.1 mg/kg, biweekly for 2 weeks in HSA^LR^ mice) - TG (oral gavage or i.p., 0.1 mg/kg, biweekly for 1 week in DMSXL mice)	HSA^LR^	- Restores cyclin D3/cdk4–CELF1 axis by downregulating CELF1 misregulation	- Improves skeletal muscle histology (central nucleation) - Improves muscle strength - Improves myotonia, muscle atrophy, and splicing defects - Reverses 17% of misregulated transcripts in the GAS of HSA^LR^ mice - Reduces CUG^exp^ transcripts	(70, 72, 75)
		DMSXL		- Attenuates anxiety-like behavior; alleviates muscle histopathology and muscle strength and CNS function	
Akt-mTOR	Inhibitors: - Rapamycin (i.p., 4 mg/kg for 1 day or 2 mg/kg for 7 or 10 days)	HSA^LR^	- No splicing correction	- Improves muscle function	(84)
AMPK	Activators: - AICAR (i.p., 500 mg/kg/d, 7 days or 6 weeks) - RSV (chow, 100 mg/kg/day, 6 weeks) - Metformin (oral gavage, 300 mg/kg, 5 or 10 days) - MK-8722 (s.c., 30 mg/kg/d, 1 or 4 weeks) - Exercise (voluntary wheel running for 8 weeks or swimming combined with AICAR for 4 weeks)	HSA^LR^	- Reduces RNA foci and MBNL1 sequestration - Induces oxidative metabolism - Corrects CELF1, Staufen1, RBM3 - Splicing correction	- Reduces central nucleation - Improves histology - Alleviates myotonia - Shift towards slow, more oxidative fibers	(84, 100, 101, 106, 107, 108, 109, 110)
TWEAK-Fn14- NF-κB	Blocking antibodies: - Anti-TWEAK (i.p., 30 μg/g/3d, 2 weeks)	DM5	- Downregulates NF-κB signaling - Downregulates atrophy gene expression	- Improves muscle histology - Improves muscle strength - Improves survival	(121)
Cn-NFAT	Inhibitor: - CsA (s.c., 25 mg/kg/day, 4 weeks)	HSA^LR^	- Increases CELF1 levels - Exacerbation of NFAT inhibition and splicing defects	- Worsens muscle histology	(139)

i.p., intraperitoneal injection; s.c., subcutaneous injection.

However, Ro-31-8220 is not entirely selective and can inhibit other kinases, including glycogen synthase kinase 3β (GSK-3β) (see below) (65). Moreover, it was shown that Ro-31-8220 eliminates nuclear foci, decreases misspliced *ATP2A1* transcripts (that encode SERCA1), and reduces the mislocalization of MBNL1 and CELF1 in a PKC-independent manner in DM1 patient-derived cells as well as in a zebrafish model expressing (CUG)^140^ transcripts. Therefore, some of its observed effects may be independent of PKC or CELF1. Indeed, other PKC inhibitors, such as GF 109203X, only showed mild effects on CELF1 levels in both cultured DM1 cells and the zebrafish model (66). Interestingly, Ro-31-8220 also improved MBNL1 localization and corrected MBNL1-dependent splicing events, such as *SERCA1*, in both DM1-derived cells (66) and HEK293 cells (61), indicating that MBNL1 may also be involved in this regulation.

Beyond PKCs, other kinases also regulate CELF1. For instance, Akt phosphorylates CELF1 at Ser-28 (67), while cyclin D3–cdk4 phosphorylates it at Ser-302, influencing its nucleocytoplasmic localization and RNA-binding activity (38, 68). These findings suggest that multiple signaling pathways converge to modulate CELF1 levels, phosphorylation status, and function. In summary, while PKC-independent mechanisms may contribute to CELF1 dysregulation in DM1, substantial evidence supports a key role for PKCs in disease pathogenesis. Targeting PKC signaling thus represents a promising avenue for therapeutic intervention in DM1. Nonetheless, many of these studies are older and depict an incomplete picture of the impact of PKC in DM1. Accordingly, this highlights the need for further investigation into the role and therapeutic potential of PKC in DM1.

### Metabolism and growth in DM1: the GSK-3β—Akt-mTOR— AMPK axis

#### GSK-3β

GSK-3β is a serine/threonine kinase with distinctive regulatory properties (Fig. 1*C*). Unlike most kinases, GSK-3β exhibits constitutive but partial activity in its basal state (69). Its full activation is mediated by phosphorylation at Tyr-216 and Tyr-279, while inhibition is achieved through phosphorylation at Ser-9 and Ser-21. GSK-3β activity is primarily regulated by the PI3K–Akt–mechanistic Target of Rapamycin (mTOR) pathway and the Wnt signaling pathway (69).

In DM1, GSK-3β activity is aberrantly regulated. Specifically, phosphorylation at Ser-9 (inactive form) is decreased, while phosphorylation at Tyr-216 (active form) is increased in skeletal muscle biopsies from DM1 patients (70). Interestingly, the inactive form was also decreased in neural stem cells derived from DM1 patients (71). These results suggest constitutive overactivation of GSK-3β in disease tissues. An increase in total GSK-3β protein levels has also been reported in muscles from DM1 patients, along with elevated phospho-Tyr-216 and reduced phospho-Ser-9 levels (70). These findings have been corroborated in HSA^LR^ (47, 70) and DMSXL mouse models (a transgenic DM1 mouse model carrying a mutated human *DMPK* gene with more than 1000 CTG repeats) (72), as well as in DM1 neuronal cultures (71) and brain tissues from DMSXL mice (72) (Table 1).

The mechanism underlying GSK-3β upregulation in DM1 remains to be fully elucidated but is clearly induced by the expression of CUG^exp^ mRNAs both *in vivo* (47, 70, 72) and *in vitro* (73). One key consequence of GSK-3β activation is its impact on RBPs, particularly CELF1. While CELF1 is known for its role in regulating alternative splicing in the nucleus, it also modulates mRNA translation and stability in the cytoplasm. Its activity is regulated by phosphorylation at Ser-302 by the cyclin D3–cdk4 complex (38). In DM1, GSK-3β hyperactivation promotes phosphorylation and subsequent degradation of cyclin D3, thereby reducing cdk4 activity. This impairs CELF1 phosphorylation at Ser-302, resulting in the accumulation of inactive CELF1 which binds to inactive eIF2α in SGs to suppress translation (38). Consequently, RNA metabolism, including alternative splicing, RNA stability, and translation, is disrupted, contributing to the pathophysiology of DM1. In this context, Ser-302A knock-in mice (CELF1-KI), which express a form of CELF1 that cannot be phosphorylated at Ser-302, exhibited diminished stereotypic behaviors and reduced white matter integrity in the CNS similar to DMSXL mice (74).

The pharmacological inhibition of GSK-3β has shown therapeutic promise in DM1. Several small molecule inhibitors, such as lithium (intervention period for 2 weeks), TDZD-8 (treatment period for 2 or 7 days), indirubin and its derivatives, 6-Bromoindirubin-3′-oxime or BIO, have been used in preclinical studies (Table 3) (70, 72, 75). These compounds restore the GSK-3β–cyclin D3 pathway, correct CELF1 misregulation, and alleviate disease features including muscle atrophy, central nucleation, myotonia, and splicing defects in HSA^LR^ mice (70, 72, 75). Another GSK-3β inhibitor, Tideglusib (TG), downregulates CUG^exp^ transcripts in DM1 myoblasts and mouse tissues from HSA^LR^ (course of treatment is biweekly for 2 weeks) and DMSXL mice (treated biweekly for 1 week) (72) and rescues approximately 17% of the misregulated transcripts in the gastrocnemius muscle of HSA^LR^ mice (74). This includes key transcripts involved in muscle regeneration, such as *Fzd7*, a receptor in the Wnt/β-catenin pathway. TG administration in DMSXL mice has also produced notable improvements in muscle histopathology, muscle strength, and CNS function (72). In juvenile DMSXL mice, biweekly injections of TG led to reduced muscle atrophy and increased grip strength (72). Cognitive benefits have also been reported, including the amelioration of anxiety-like behavior in prenatally treated DMSXL mice (72). These effects coincided with restored active CELF1 in the brain, highlighting a CNS-relevant role of CELF1 misregulation (72, 74). Crucially, the therapeutic relevance of GSK-3β inhibition has advanced into the clinic. A phase II clinical trial in adolescent and adult patients with congenital and childhood-onset DM1 has demonstrated that GSK-3β inhibition with TG yields promising results, improving both muscle and CNS outcomes (76).

Together, the preclinical and clinical data indicate that correcting GSK-3β activity restores essential biological processes disrupted by toxic CUG repeats, positioning the GSK-3β–CELF1 axis as a highly attractive therapeutic target in DM1. To date, research on GSK-3β has predominantly focused on its regulation of CELF1 *via* the cyclin D3–cdk4 axis. However, with over 100 known substrates, it is likely that GSK-3β affects many additional pathways relevant to DM1 pathogenesis.

#### Akt-mTOR signaling

mTOR (mechanistic Target of Rapamycin) is a serine/threonine kinase that plays a critical role in the regulation of protein synthesis and degradation (77, 78) (Fig. 1*D*). As such, it is a key modulator of skeletal muscle mass by balancing hypertrophy and atrophy (77, 78, 79). mTOR functions as a part of two distinct multiprotein complexes: mTORC1 and mTORC2. Of these, mTORC1, which is comprised of mTOR and Raptor, is primarily responsible for promoting muscle protein synthesis and cell growth (77, 78). One of its main upstream activators is Akt or protein kinase B, a serine/threonine kinase involved in cell survival, proliferation, and glucose metabolism (77, 78). Upon activation, Akt promotes mTOR activity by phosphorylating it at Ser-2448 and by inhibiting the TSC1/2 complex, which normally suppresses mTORC1 *via* the GTPase Rheb (77, 78, 80). Once activated, mTORC1 enhances protein synthesis through the phosphorylation of ribosomal protein S6 kinase (p70S6K) at Thr-389 and 4E-binding protein 1, leading to the release of ribosomal protein S6 and eIF4E. This then facilitates the translation of mRNAs associated with cell proliferation and growth (77, 78, 79, 81).

Studies examining the Akt–mTOR pathway in DM1 have yielded inconsistent results. Akt protein levels are highly variable in patient-derived DM1 myotubes (82). mTOR phosphorylation at Ser-2448 is decreased in both human and iPSC-derived satellite muscle cells (83), suggesting reduced mTOR activity. In contrast, while Akt phosphorylation remains unchanged in cultured DM1 neural cells, ribosomal protein S6 phosphorylation is decreased (71). In muscle biopsies from DM1 patients, Akt phosphorylation appears unaffected, but elevated levels of phosphorylated p70S6K and S6 have been reported (84). Similarly, in HSA^LR^ mice, Akt and mTOR levels are unchanged, yet increased phosphorylation of ribosomal S6 protein is observed (84). These findings suggest that despite potential upstream impairment, the downstream activation of p70S6K and S6 may partially sustain protein synthesis in DM1 tissues (Table 1).

The Akt–mTOR axis also plays a central role in the regulation of autophagy. Under conditions where mTOR is repressed, autophagy is triggered by AMP-activated protein kinase (AMPK)-dependent activation of ULK1, leading to the formation of the phagophore (84, 85). Subsequent steps involve the ATG5–ATG12 conjugate and conversion of LC3-I to LC3-II, a hallmark of autophagosome formation (86). Elevated levels of autophagy markers, ATG5, ATG12, LC3-II, and the autophagosome cargo protein p62, have been observed in differentiating DM1 myoblasts (87), DM1 neural cells (71), muscle satellite cells (83), and in HSA^LR^ mouse muscle (84). In addition, the accumulation of autophagic vacuoles has been reported in biopsies from DM1 patients (87), HSA^LR^ mice (84), and *Drosophila* DM1 models (88), indicating increased autophagic activity. Interestingly, pharmacological inhibition of autophagy using chloroquine improves phenotypic features in DM1 fly models and HSA^LR^ mice (88), including MBNL1-regulated splicing and myotonia. Overexpression of MBNL1 in fly and mouse models reduces autophagy, likely through mTOR activation, further supporting the regulatory role of MBNL1 in autophagic pathways (83). Furthermore, recent studies have shown that Staufen1 overexpression is associated with mTOR activation and regulation of autophagy (89, 90). It is therefore plausible that the Staufen1 increase contributes to the regulation of mTOR and autophagy in DM1. Beyond mTOR signaling, other pathways can also regulate autophagy. Indeed, AMPK inhibits mTORC1 by phosphorylating TSC2 or Raptor and ULK1, thereby initiating autophagosome formation and also, similarly, PKR activation and eIF2α control autophagy (85). It is therefore likely that the combined dysregulation of signaling pathways impacts autophagic processes in DM1.

DM1 is characterized by muscle fiber size variability and selective atrophy of type I fibers (91, 92). While muscle atrophy has been recapitulated in several DM1 models including DM300 transgenic mice (93), inducible EpA960/HSA-Cre-ER^T2^ (56), TREDT960I mice (or CUG960) (94), and DM1 *Drosophila* (88), it is absent in the widely used HSA^LR^ mouse model (34, 95). Muscle atrophy is typically regulated *via* Akt–mTOR suppression of the FoxO transcription factors, which induce the expression of atrophy-related genes or atrogenes, such as *Atrogin-1/MAFbx* and *MuRF1* (78). However, in DM1 myotubes (82), no clear induction of these E3 ligases is observed. An increase in Atrogin-1, but not MuRF1, has been detected in DM300 mice (96), leaving the contribution of the Akt–mTOR–FoxO axis to DM1-associated muscle atrophy uncertain.

Alternative mechanisms likely contribute to muscle wasting in DM1. Overexpression of CELF1 in transgenic mice induces muscle atrophy (97), particularly when targeted to myonuclei (98). Likewise, sustained expression of Staufen1 in skeletal muscle results in progressive myopathy with pronounced histological and functional deficits. This phenotype is associated with the repression of PI3K–Akt signaling and induction of FoxO3a, MuRF1, and Atrogin-1 (46). Further increasing Staufen1 expression in HSA^LR^ mice (*via* AAV or transgenic approaches) induces muscle atrophy, mimicking more severe forms of the disease (47).

Interestingly, treatment with rapamycin, a selective mTOR inhibitor, improves muscle function in HSA^LR^ mice without correcting alternative splicing defects (84) (Table 3). These findings indicate that although the exact status of Akt–mTOR signaling in DM1 remains inconclusive, the downstream autophagy pathway is consistently activated, while the canonical atrophy pathway (FoxO-mediated transcription of atrogenes) appears unaffected.

#### AMPK signaling

AMPK is a central regulator of cellular energy homeostasis and an emerging mediator of mitochondrial biogenesis and skeletal muscle plasticity (Fig. 1*E*). AMPK functions as a heterotrimeric complex consisting of one catalytic α subunit and two regulatory β and γ subunits (for review, see (99)). In skeletal muscle, the predominant isoform is the α2β2γ1 complex. AMPK is activated by upstream kinases such as LKB1 and CaMKKβ and regulates several transcriptional co-activators, including peroxisome proliferator-activated receptor gamma coactivator-1 alpha (PGC-1α), which promotes mitochondrial biogenesis. As such, AMPK serves as both an energy sensor and a master regulator of cellular metabolism in skeletal muscle.

As with Akt/mTOR signaling, conflicting data have been reported regarding the status of AMPK signaling in DM1 muscle (Table 1). In a recent study, our laboratory observed a significant reduction in AMPK activity in HSA^LR^ mice, as evidenced by decreased phospho-AMPK/AMPK ratios (100). These findings contrast with earlier reports showing either i) no alteration in AMPK signaling in DM1 neuronal cells (71) and skeletal muscle from HSA^LR^ mice (although AMPK signaling was shown to be unresponsive to metabolic stress under fasting conditions (84)) or ii) an increase in the inducible CUG960 mouse model (94). This discrepancy highlights the need for a systematic and comprehensive analysis of AMPK and Akt/mTOR signaling pathways in DM1, accounting for variables such as age, sex, muscle fiber type (slow- vs. fast-twitch), and diet (see Cumulative impact of disrupted signaling pathways in DM1 skeletal muscle).

Despite these uncertainties, accumulating evidence supports a beneficial role for AMPK activation in DM1 muscle (Table 3). Pharmacological activation of AMPK using 5-Aminoimidazole-4-carboxamide-1-β-D-ribofuranoside (AICAR), a synthetic pan AMPK activator, has been shown to ameliorate several hallmarks of RNA toxicity, including the reduction of CUG^exp^ RNA aggregates, correction of alternative splicing defects, and mitigation of myotonia in HSA^LR^ mice (84, 100, 101). Furthermore, extended AICAR treatment (daily injections with AICAR for 6 weeks) enhances muscle histology by promoting fiber type transitions, oxidative metabolism, reduced fiber size variability, and decreased central nucleation, suggesting time-dependent therapeutic effects (100). Resveratrol, which activates AMPK signaling indirectly *via* SIRT1, has also been shown to rescue splicing defects in HSA^LR^ mice (100). Similar effects have been reported in DM1 fibroblasts and myotubes, where resveratrol corrected alternative splicing of *INSR* and *RYR1* (102, 103). Consistent with the rescue of *RYR1* splicing, calcium signaling was corrected in DM1 myotubes in culture (102). Metformin, a widely used antidiabetic drug and AMPK activator, was shown to correct splicing abnormalities in DM1 patient-derived human embryonic stem cell lines (hESCs) and myoblasts (104). However, in contrast to AICAR, a high-dose of metformin failed to improve splicing defects in HSA^LR^ mice (84). Encouragingly, a small-scale phase II clinical trial reported that metformin improved mobility and gait parameters in DM1 patients (105). Although AICAR and metformin show therapeutic potential in DM1, their likely AMP-independent effects have prompted interest in examining the therapeutic potential of more selective AMPK activators. MK-8722, a direct allosteric activator targeting the drug and metabolite site located between the α and β subunits of the AMPK complex, has shown efficacy in skeletal muscle. Our laboratory recently demonstrated that short- and long-term MK-8722 treatments improved splicing defects and promoted a shift toward oxidative fibers in DM1 mice. More specifically, MK-8722 improved *Atp2a1* and *Bin1* splicing in the muscles of male HSA^LR^ mice relative to their vehicle counterparts. Moreover, MK-8722 treatment reduced the percentage of central nucleation toward WT levels as well as induced a decrease in the proportion of larger muscle fibers. Results also showed an increased proportion of type IIB fibers in the muscle of female DM1 mice (106).

Physiological activation of AMPK signaling, particularly through exercise, has also been investigated in DM1. Voluntary wheel running improved the spliceopathy in HSA^LR^ mice, and correction of *CLCN1* splicing was associated with improved myotonia (100, 107, 108, 109). Our lab also examined a 4-weeks combinatorial treatment with exercise and AICAR (110). This approach significantly improved muscle pathology, with stronger effects in female DM1 mice, including reduced RNA foci, improved splicing, increased fiber size, and a shift toward oxidative fibers. These data reinforce the therapeutic potential of AMPK activation *via* both pharmacological and physiological means, while also highlighting sex-specific effects of AMPK activators and exercise (111). In DM1 patients, exercise was also shown to improve disease outcomes (112, 113) and improve aberrant alternative splicing (114).

GSK-3β can directly phosphorylate AMPKα on residues that interfere with upstream activation by LKB1, thereby preventing phosphorylation at Thr-172 and suppressing AMPK activation (115). Conversely, AMPK activation can indirectly regulate GSK-3β activity (116). Thus, GSK-3β upregulation observed in DM1 muscle may contribute to impaired AMPK signaling. Accordingly, therapeutic strategies aimed at activating AMPK could lead to AMPK-dependent beneficial effects but also mitigate GSK-3β activity in DM1 skeletal muscle.

In conclusion, AMPK appears to play an important role in modulating the DM1 pathology. Whether activated pharmacologically or physiologically, AMPK signaling ameliorates molecular, biochemical, and histological features of DM1, positioning it as a promising therapeutic target. Although the beneficial effects seen with AMPK activators may not entirely function in an AMPK-dependent manner, their reliance on AMPK signaling further highlights this pathway as a highly relevant therapeutic target that warrants deeper mechanistic and translational investigation. Continued investigation into selective activators and sex-specific responses, as well as genetic deletion approaches will be essential to fully harness the role and therapeutic potential of AMPK in DM1.

### Inflammation-driven fibrosis in DM1 skeletal muscle

#### TWEAK-Fn14 and NF-κB signaling

Fibroblast growth factor-inducible 14 (Fn14) is a member of the tumor necrosis factor (TNF) receptor superfamily and serves as the receptor for TNF-like weak inducer of apoptosis (TWEAK) (Fig. 1*F*). Under normal physiological conditions, adult skeletal muscle expresses very low levels of Fn14. However, Fn14 expression is markedly upregulated in response to various stressors such as starvation, denervation, or immobilization. Upon binding to its ligand, the TWEAK–Fn14 axis activates both the canonical (classical) and non-canonical (alternative) NF-κB signaling pathways, leading to muscle atrophy, fiber type switching, and metabolic remodeling (for review, see (117, 118)).

The NF-κB transcription factor family includes five members: p105/p50 (NF-κB1), p100/p52 (NF-κB2), p65/RelA, RelB, and c-Rel. Activation of the canonical NF-κB pathway by the TWEAK–Fn14 interaction involves the recruitment of TRAF proteins and activation of the TAK1 kinase, which in turn stimulates the IκB kinase (IKK) complex (comprising IKKα, IKKβ, and IKKγ) (119, 120). This leads to phosphorylation and proteasomal degradation of IκBα, a cytoplasmic inhibitor of NF-κB. The degradation of IκBα allows p65/RelA–p50 dimers to translocate into the nucleus and activate genes involved in inflammation and muscle atrophy. In parallel, the non-canonical NF-κB pathway is activated by stabilization and accumulation of NF-κB–inducing kinase, which selectively phosphorylates and activates IKKα. This triggers the processing of p100 into p52, enabling the formation of p52–RelB dimers that translocate to the nucleus and drive gene expression involved in metabolic reprogramming and muscle remodeling.

In the context of DM1, Fn14 is upregulated at both the mRNA and protein levels in skeletal and cardiac muscle tissues from inducible DM5 and DM200 mouse models (Table 1), but not in the HSA^LR^ model, highlighting intrinsic differences among DM1 mouse models (121). Elevated Fn14 expression has also been observed in muscle biopsies from DM1 patients (121). Furthermore, increased phosphorylation of p65/RelA and enhanced processing of p100 into p52 suggest that both the canonical and non-canonical NF-κB pathways are activated in DM1 skeletal muscle, thereby facilitating fibrosis, inflammation, and atrophy (121).

Genetic deletion of Fn14 in DM5 mouse models (DM5/Fn14^−/−^ mice) or treatment with anti-TWEAK blocking antibodies significantly reduced muscle pathology and improved muscle function, without correcting features such as myotonia, cardiac conduction defects, or aberrant splicing (121) (Tables 2 and 3). Interestingly, *TWEAK* transcript levels were not altered in either DM1 mouse models or patient tissues (121).

Nevertheless, TWEAK activation exacerbates the pathology while genetic deletion of TWEAK (DM5/Tweak^−/−^) ameliorated muscle histology and improved functional outcomes, further confirming that TWEAK–Fn14 signaling contributes to disease progression through NF-κB–mediated mechanisms (122) (Table 2). These findings demonstrate that TWEAK–Fn14 activation and downstream NF-κB signaling exacerbate skeletal muscle pathology in DM1 independently of RNA splicing abnormalities.

Beyond TWEAK–Fn14, several other pathways implicated in DM1 also converge on NF-κB activation. These include PKR (123, 124, 125), PKC (126, 127), GSK-3β (128, 129, 130), and the Akt–mTOR axis (131, 132, 133), which are also known to activate NF-κB in various cell lines. It is therefore likely that these pathways collectively converge towards and contribute to NF-κB activation, which ultimately affect muscle function and histology in DM1 context.

### Defective fiber remodeling and regeneration in DM1

#### Calcineurin–NFAT signaling

Calcineurin (Cn) is a calcium/calmodulin-dependent serine/threonine phosphatase that plays a pivotal role in skeletal muscle adaptation and disease processes (134). It is a heterodimer composed of a catalytic subunit (CnA) and a regulatory subunit (CnB) (134) (Fig. 1*G*). Cn is activated in response to intracellular calcium fluctuations through its interaction with calmodulin (135). Once activated, CnA stimulates the activity of nuclear factor of activated T-cell (NFAT) transcription factors through dephosphorylation and translocation into nuclei, where they activate transcription of target genes (134, 136, 137). In skeletal muscle, NFAT activity induced by Cn promotes the expression of a slow, oxidative myogenic gene program (136) and contributes to the regulation of muscle fiber size (134, 138).

Work from our laboratory demonstrated that Cn signaling is upregulated and activated in HSA^LR^ mice (139) (Table 1). Notably, the expression levels of endogenous inhibitors of the pathway, such as Calsarcin-2 and RCAN1.1, remain unchanged, whereas the expression of key activators, including muscle LIM protein and four and a half LIM domains 1 (FHL1), is increased. Consequently, NFATc1 is both upregulated and redistributed to the nucleus in DM1 myonuclei, accompanied by the increased expression of canonical NFAT targets, such as RCAN1.4 and myoglobin (139). These changes are associated with a redder appearance of extensor digitorum longus muscles in HSA^LR^ mice, consistent with a shift toward oxidative metabolism (139). Pharmacological inhibition of Cn with cyclosporin A exacerbates the disease phenotype in HSA^LR^ mice (Table 3), supporting the notion that upregulated Cn–NFAT signaling represents a compensatory, protective adaptation in DM1 skeletal muscle (139).

In addition to the Cn pathway, several other signaling cascades converge on NFAT. PKC-θ cooperates with CnA to activate both NFAT and myocyte enhancer factor-2, promoting slow-twitch muscle gene expression in cultured myogenic cells (140). Similarly, TWEAK acting *via* its receptor Fn14 can modulate genes such as *PGC-1α*, further contributing to the oxidative phenotype (141). Conversely, active GSK-3β antagonizes NFAT nuclear localization and may counteract the combined effects of these signaling pathways (142). Together, these findings highlight the intricate regulation of NFAT signaling in DM1 and suggest that sustained activation of Cn–NFAT may serve as a beneficial response to the underlying pathology in DM1 skeletal muscle.

### Emerging omics insights into DM1 signaling defects

In addition to pathway-specific studies, recent omics approaches have provided new insights into the impact of disrupted signaling pathways underlying the DM1 pathology (see (143)). In this context, both transcriptomic (for example (112, 114, 144, 145, 146, 147)) and proteomic (for example (146, 148, 149)) approaches have been employed and several studies have indeed reported the dysregulation of signaling proteins in DM1 skeletal muscle (146, 148, 149). In particular, a quantitative proteomic analysis of muscle biopsies of the *Vastus Lateralis* muscle from 11 DM1 patients undergoing a 12-weeks strength-training program identified over 570 proteins, of which 44 were significantly expressed after this training regimen (148). Notably, six proteins, CAPN3, CSRP3, YWHAE, MYBPH, FLNC, and FHL3, emerged as markedly modulated in DM1 patients following exercise with all of them being upregulated post-training except for FHL3 which was downregulated. Collectively, these proteins are responsible for sarcomere remodeling and repair, energy metabolism, myogenic differentiation, as well as cellular stress response, and their alterations following exercise are thought to convey positive beneficial outcomes on DM1 skeletal muscle (148). Similarly, another study identified novel alternative splicing events in *FLNC* and *YWHAE* pre-mRNAs and validated their expression at the protein level in the gastrocnemius muscle of DM1 HSA^LR^ mice, following elegant, integrated transcriptomic and proteomic analyses (146).

Moreover, a recent unbiased GSK-3β–linked proteomic study conducted in DM1 patient fibroblasts reported that elevated GSK-3β activity triggered by toxic RNA foci in DM1 causes dysregulation of key downstream targets (149). Three proteins were shown to be highly dysregulated especially in cells containing a high number of CUG repeats in a GSK-3β–dependent manner. These included CTPS1, CAPN1, and HDAC2. CTPS1 is linked to changes in metabolism, while CAPN1 is expressed ubiquitously and is involved in cytoskeletal remodeling, signal transduction, and apoptosis. Finally, HDAC2 regulates cell growth and apoptosis. Importantly, and in the context of the current discussion, these proteins were also found to be dysregulated in other DM1 cell types, including skeletal muscle, raising the possibility that these proteins have a multisystemic impact.

To complement proteomics-based studies in DM1, detailed transcriptomic analyses have also been conducted to better characterize differential gene expression and missplicing using RNA-seq (144, 145). In this work, it was found that the Akt and PLEKHG5 signaling pathways were dysregulated in DM1 patient myoblasts and myotubes, respectively, with the latter being known to activate NF-κB signaling (144). Moreover, a comprehensive transcriptomic study examined healthy and DM1 hESCs to gain insight into the initial steps of early myogenic differentiation in DM1 (145). Their results revealed that during the differentiation of hESCs into myoblasts, TNFα signaling *via* NF-κB was stimulated. However, mTOR signaling, an important pathway in myogenesis, was suppressed in DM1 samples during the transition from myoblasts to myotubes, further highlighting that mTOR signaling is abnormally activated in the DM1 context.

A computational text-mining and enrichment analysis was also conducted on bibliometric analysis, including articles combining DM1 and metabolic/metabolism terms to elucidate metabolism-related pathways linked to DM1. Seventy one genes not previously associated with DM1 were identified, with a strong enrichment in metabolic and signaling processes, including the PI3K-Akt activation. Additionally, valuable druggable candidates that are related to metabolic processes in DM1 were revealed. These included proteins involved in metabolic regulation, cytoskeletal stability, RNA processing, skeletal muscle fiber adaptation and development, apoptosis, and inflammation (147).

Collectively, these omics studies provide comprehensive insight into pathway-focused therapeutic targets, revealing interconnected alterations in many of the pathways discussed above. This indicates that many of the cellular and molecular processes that underly DM1 cannot be solely understood from a single-pathway analysis. By identifying biomarkers and therapeutic targets associated with these metabolic processes in a systematic approach, it will more likely lead to an effective and multifaceted therapeutic approach with impact that will circumvent key aspects of the DM1 pathology.

## Cumulative impact of disrupted signaling pathways in DM1 skeletal muscle

**Figure 2:**
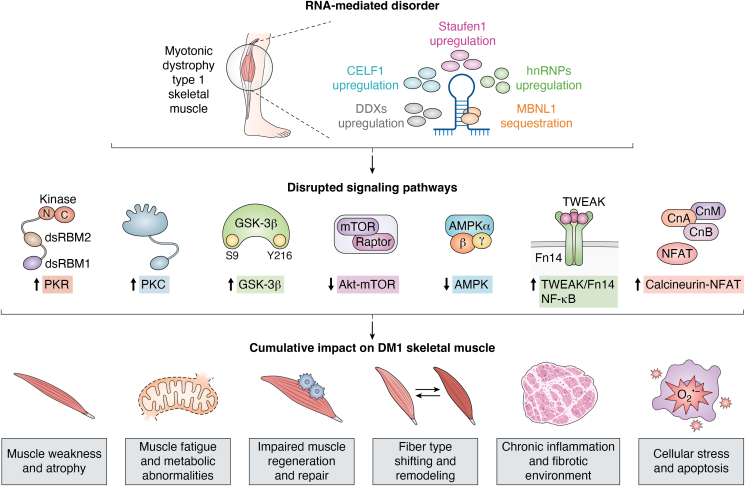
**Visual abstract of the cumulative impact of the disruptions in signaling pathways in DM1 skeletal muscle**.

Based on the evidence presented above, it seems clear that a complex network of signaling pathways is dysregulated in DM1. These pathways impair diverse muscle functions and processes that are essential for maintaining muscle homeostasis, including regulating protein synthesis and degradation, muscle repair, mitochondrial biogenesis, energy metabolism, and inflammation (Fig. 2) (150, 151, 152, 153). However, the cumulative impact of these disruptions remains difficult to establish given that in most instances, these dysregulated pathways are examined individually and within specific experimental contexts, for example, different mouse models, muscle type, age, sex, various cell types, …etc, that vary across studies (1, 26). Additionally, the nature of the disruptions themselves with, for example, increases or decreases in signaling that could cause compensatory benefits or detrimental effects, respectively, further clouds the overall picture. Finally, and as discussed in the above sections, a substantive amount of signaling cross-talks takes place across these pathways (115, 131, 132, 133). Nonetheless, it seems reasonable to postulate that the combination of signaling defects leads to an additive (or perhaps even synergistic), detrimental cascade of effects. Moreover, the interactions and cross-talks between these disrupted pathways may vary over time and across muscle types thereby impacting disease severity (91, 92, 114). Accordingly, this makes it further difficult to precisely define their joint impact. While many studies assess individual pathways in isolation, our review emphasizes that it is the cumulative disruption of Akt–mTOR, GSK-3β, AMPK, PKC, PKR, TWEAK–Fn14, and Cn that explain the broader, multisystemic clinical manifestations of DM1. This includes muscle weakness and atrophy, muscle fatigue and metabolic abnormalities, as well as impaired muscle regeneration and repair, perturbed fiber type remodeling, and chronic inflammation and apoptosis (34, 82, 87, 88, 94, 96, 119). We propose that these phenotypes cannot be understood through single-pathway analyses but emerge from interconnected signaling crosstalk, which over time exacerbates muscle dysfunction in a progressive and systemic manner. Thus, one can envisage that the following phenotypic characteristics of skeletal muscle and its disease progression are affected by the cumulative effects of disrupted signaling pathways.

### Muscle weakness and atrophy

Several of the key signaling pathways that are disrupted in DM1 muscle cause weakness, atrophy, and wasting. In particular, the Akt–mTOR pathway is essential for muscle growth given that it stimulates protein synthesis. However, its downregulation in DM1 impairs muscle hypertrophy, promotes protein breakdown, and contributes to atrophy (71, 78, 79, 81, 84). GSK-3β is normally suppressed by Akt, but when Akt is inhibited, GSK-3β becomes overactive, further suppressing protein synthesis and promoting muscle protein degradation (69, 70, 75). The TWEAK–Fn14 pathway is another critical factor in muscle atrophy, with chronic activation of TWEAK through its receptor Fn14, promoting muscle catabolism (117, 118, 121). In DM1, this latter pathway is upregulated, leading to further muscle protein breakdown through the ubiquitin-proteasome system and autophagy, ultimately reducing muscle mass and function (121, 141). TWEAK–Fn14 also induces the expression of atrogenes such as *MuRF1* and *Atrogin-1*, *via* NF-κB activation, which promotes additional muscle wasting (117, 119, 141). Together, these disruptions can cause severe muscle wasting, especially in distal muscles like those of the hands, forearms, and lower legs, which is characteristic of DM1 (1). Additionally, PKR, activated by interferon signaling, contributes to muscle protein synthesis and degradation, and its disruption further impairs muscle maintenance, promoting muscle wasting (31, 123, 124). The combined effects of these disrupted pathways result in progressive muscle atrophy and an increase in the expression of atrophy-related genes, ultimately exacerbating the muscle weakness seen in DM1 (94, 96).

### Muscle fatigue and metabolic abnormalities

Several factors contribute to muscle fatigue, energy deficits, and metabolic abnormalities in DM1 skeletal muscle. Notably, AMPK, a master regulator of energy balance, is crucial for cells to respond to energy stress, but its dysregulation in DM1 results in an energy imbalance, reduced mitochondrial function, and compromised metabolic processes such as fatty acid oxidation and glucose uptake (84, 99, 100, 111). The dysfunction of AMPK specifically affects mitochondrial biogenesis and energy homeostasis, contributing to symptoms such as glucose intolerance, metabolic inflexibility, and increased fatigability (84, 99). This leads to inefficient energy metabolism and results in muscle fibers with poor endurance (108, 109). PKR, often activated due to stress or toxic RNA, further disrupts cellular metabolism by inhibiting mitochondrial activity (31, 38). Additional key regulators of glucose metabolism, including GSK-3β, and Akt, are also disrupted, contributing to insulin resistance, impaired glucose uptake, and mitochondrial dysfunction (9, 70, 131). Finally, TWEAK exacerbates this metabolic impairment by reducing mitochondrial content in skeletal muscle. Overall, these converging disruptions in signaling lead to fatigue, poor oxidative metabolism, and decreased muscle endurance (84, 100, 106, 111).

### Impaired muscle regeneration and repair

Several critical pathways involved in muscle regeneration and repair are affected in DM1, which can lead to long-term muscle damage. Among these, PKC and Cn are essential for activating satellite cells and promoting myogenic differentiation, but their dysregulation in DM1 could cause impaired satellite cell activation and defective muscle fiber repair (140, 134, 136, 137). TWEAK-Fn14 further suppresses myogenic differentiation and promotes fibrosis, which together can worsen muscle degeneration (117, 118). PKR, which is activated in DM1, induces cell stress responses and apoptosis, thereby exacerbating muscle damage (31). In addition, PKR activation also leads to the phosphorylation of eIF2α which shuts down global translation, further inhibiting muscle growth and repair (38, 85). Coupled to these defects, the suppression of mTOR inhibits muscle protein synthesis and myogenesis, while disrupted Cn–NFAT signaling hinders regeneration (79, 81, 139). Together, the disruption of PKC, Cn, Akt–mTOR, and other pathways leads to failures in efficient muscle regeneration and creates fiber damage and dysfunction of satellite cells. These defects collectively result in poor muscle regeneration following injury and contribute to the ongoing muscle wasting seen in DM1 (134, 139).

### Fiber type remodeling

Disruptions in Cn signaling, AMPK, PKC, and TWEAK-Fn14, are expected to jointly lead to altered muscle fiber composition and function. Cn and AMPK promote the development of slow-twitch oxidative fibers, but their dysregulation in DM1 might shift the balance towards faster fibers which are more prone to degeneration, fatigue, and atrophy (99, 135, 136). PKC isoforms, essential for maintaining muscle fiber phenotype and cytoskeletal dynamics, are also impaired in DM1 and may therefore further contribute to the transition from slow fibers to faster ones (51, 140). Such a switch is expected to be accompanied by reductions in the oxidative capacity of muscle fibers, adding to the decreased endurance (99). Finally, TWEAK-Fn14 is known to also play a role in promoting the slow-to-fast fiber type transition, further impacting overall muscle function and endurance (117). Linked to all this, the dysregulation of calcium signaling, critical for fiber type maintenance, along with disruptions in AMPK, contributes to the overall decline in normal muscle contractile properties, increasing fatigue and reducing muscle endurance in DM1 (22, 24, 139).

### Chronic inflammation and fibrosis

The TWEAK–Fn14 signaling pathway plays a critical role in promoting chronic inflammation and fibrosis which, in DM1 muscle, could further exacerbate muscle damage and impair natural muscle repair mechanisms (117, 118). TWEAK–Fn14 activates NF-κB, leading to the expression of inflammatory cytokines and the promotion of fibrosis, which ultimately shifts the muscle environment from one of regeneration to degeneration (119, 120, 141). Chronic activation of TWEAK-Fn14, along with disruptions in other pathways such as PKR, activates pro-inflammatory cascades, including interferon responses, which drive sustained inflammation, immune cell infiltration, and fat accumulation within muscle tissue (31, 123, 124, 125). This process replaces functional muscle tissue with non-functional, fibrotic, and/or adipose tissues, leading to increased muscle stiffness and a decline in overall muscle function and performance (91). The result is a cycle of chronic low-grade inflammation and fibrosis that further impairs muscle healing and contributes to the progressive decline in muscle function in DM1 (117, 121).

### Cellular stress and apoptosis

In DM1, increased cellular stress and apoptosis may be driven by several key pathways. PKR is activated by toxic dsRNAs, such as those seen with the expanded CUG repeats in *DMPK* transcripts, which triggers translational repression, phosphorylation of eIF2α, SG formation, and, ultimately, cell apoptosis (31, 33, 38, 45, 48). In addition, TWEAK–Fn14 and GSK-3β contribute to pro-apoptotic signaling, further promoting cell death (69, 117). This heightened cellular stress and apoptosis lead to muscle cells becoming more prone to death, exacerbating muscle degeneration and contributing to the progressive nature of the disease (82, 88).

## Perspective and conclusion

The full spectrum of perturbations in signaling pathways in DM1 tissues is still not fully understood. Moreover, a certain level of variability in the extent of the signaling defects in DM1 muscles has been observed, likely due to the varied experimental approaches and model systems used in different studies. In addition to the disrupted pathways discussed here, there are likely several others that are also misregulated in DM1 skeletal muscle, but their nature and impact remain to be established. The evidence reviewed here clearly highlights that the full DM1 pathophysiology cannot be accounted for by any single signaling pathway. Instead, the complexity of the disease emerges from both RNA toxicity (as mentioned above) as well as cumulative and interconnected dysregulation of multiple signaling cascades, including Akt–mTOR, GSK-3β, AMPK, PKC, PKR, Cn, and TWEAK–Fn14. When considered in isolation, each pathway contributes to discrete cellular outcomes. When these perturbations are integrated on a systematic level, they better explain the multifaceted DM1 phenotype. This cumulative impact results in a broader mechanistic integration of disrupted signaling into a coherent explanatory model for DM1 pathogenesis, thereby providing a broader framework for therapeutic interventions.

Of relevance, DMPK itself is a serine/threonine protein kinase. While its function is still not well characterized, it is known to be related to the MRCK p21-activated kinases and the Rho family of small GTPases (154). Moreover, DMPK substrates are still poorly identified. The sequestration of CUG^exp^ *DMPK* transcripts reduces DMPK protein expression through haploinsufficiency (155). It is likely, therefore, that its decreased expression also contributes to the DM1 muscle phenotype (156, 157).

One key question is how mechanistically CUG^exp^ expression causes the misregulation of all these signaling pathways and whether these occur through direct mechanisms or *via* secondary events. In this context, global changes in alternative splicing are observed in DM1 (158). It is therefore likely that several transcripts encoding proteins involved in signaling pathways are misspliced in DM1, altering the properties and functions of signaling molecules and the overall status of specific pathways. The alternative splicing status of these signaling molecules has yet to be systematically explored and a thorough analysis of splicing isoforms of each signaling molecule described in this review should provide key insight into the molecular basis underlying at least some of the signaling disruptions. This clearly highlights the need for comprehensive and systematic analyses of the status of these pathways and how they are interconnected in DM1.

The fact that the modulation of these signaling pathways by pharmacological molecules and transgenic approaches in cell systems and animal models has corrected defects associated with DM1 represents a major advance in the comprehension of the DM1 pathomechanism and for the development of novel therapeutic strategies (64, 70, 84, 100, 110, 121, 122, 139). This also indicates that combinatorial therapies targeting simultaneously multiple signaling pathways and/or events associated with RNA toxicity (RNA sequestration, RBP sequestration and misregulation, alternative splicing) might be necessary to fully correct the multitude of defects observed in DM1 skeletal muscle.

Several testable therapeutic approaches emerge from this review. For example, the combination of Akt–mTOR inhibition and AMPK activation to stimulate protein synthesis and mitochondrial biogenesis while simultaneously improving muscle atrophy and fatigue may prove very attractive for optimized effects on DM1 skeletal muscle. Similarly, inhibiting TWEAK–Fn14 signaling in conjunction with Cn activation may limit inflammation and fibrosis and induce a slower and more oxidative myofiber program with therapeutic benefits including enhancing muscle repair. Additionally, targeting PKR and/or TWEAK–Fn14 in combination with AMPK or Akt–mTOR may relieve translational repression, cellular apoptosis, as well as restore growth signaling to improve several DM1 histopathological features. While these therapeutic combinations are hypothetical at present and will first require rigorous preclinical validation, such novel and timely approaches may prove ideal for improving the multiple symptoms characteristic of DM1 skeletal muscle.

In conclusion, the combined impact of dysregulated signaling pathways broadly affects multiple key aspects of DM1 muscle functions and characteristics. Acting together, these disrupted signaling cascades contribute profoundly to the hallmark symptoms of DM1. Together with the splicing abnormalities, these signaling defects drive the characteristic muscle weakness, fatigue, metabolic defects, and regeneration failure seen in DM1. Based on the importance and impact of these signaling pathways on multiple aspects of the DM1 muscle phenotype, it appears that therapeutically targeting these disruptions *via* a multi-target approach centered on the cumulative and interconnected nature of these signaling disruptions is a critical area for additional research. This approach may support the quest to slow or reverse muscle dysfunction in DM1 and to improve the quality of life of DM1 patients.

## Declaration of Generative AI and AI-Assisted Technologies in the Writing Process

During the preparation of this work, the author(s) used “ChatGPT” in order to generate content related to Cumulative impact of disrupted signaling pathways in DM1 skeletal muscle of this review. After using this tool/service, the author(s) reviewed and edited the content as needed and take(s) full responsibility for the content of the publication.

## Conflict of interest

The authors declare that they have no conflicts of interest with the contents of this article.
